# Using deep-learning predictions reveals a large number of register errors in PDB depositions

**DOI:** 10.1107/S2052252524009114

**Published:** 2024-10-10

**Authors:** Filomeno Sánchez Rodríguez, Adam J. Simpkin, Grzegorz Chojnowski, Ronan M. Keegan, Daniel J. Rigden

**Affiliations:** a Institute of Systems, Molecular and Integrative Biology University of Liverpool LiverpoolL69 7ZB United Kingdom; b Life Science Diamond Light Source Harwell Science and Innovation Campus DidcotOX11 0DE United Kingdom; c Department of Chemistry, York Structural Biology Laboratory University of York York United Kingdom; d European Molecular Biology Laboratory Hamburg Unit, Notkestrasse 85 22607Hamburg Germany; e UKRI–STFC Rutherford Appleton Laboratory Research Complex at Harwell DidcotOX11 0FA United Kingdom; Chinese Academy of Sciences, China

**Keywords:** deep learning, register errors, Protein Data Bank, structure validation, *AlphaFold*2

## Abstract

The accuracy of the information in the Protein Data Bank (PDB) is of great importance for the myriad downstream applications that make use of protein structural information. Despite best efforts, the occasional introduction of errors is inevitable, especially where the experimental data are of limited resolution. A novel protein structure validation approach based on spotting inconsistencies between the residue contacts and distances observed in a structural model and those computationally predicted by methods such as *AlphaFold*2 has previously been established. It is particularly well suited to the detection of register errors. Importantly, this new approach is orthogonal to traditional methods based on stereochemistry or map–model agreement, and is resolution independent. Here, thousands of likely register errors are identified by scanning 3–5 Å resolution structures in the PDB. Unlike most methods, the application of this approach yields suggested corrections to the register of affected regions, which it is shown, even by limited implementation, lead to improved refinement statistics in the vast majority of cases. A few limitations and confounding factors such as fold-switching proteins are characterized, but this approach is expected to have broad application in spotting potential issues in current accessions and, through its implementation and distribution in *CCP*4, helping to ensure the accuracy of future depositions.

## Abstract 2

A novel structure-validation method is applied to PDB depositions at 3–5 Å resolution, revealing large numbers of putative register errors.

## 1. Introduction

For more than five decades, the Protein Data Bank (PDB; wwPDB Consortium, 2019 ▸) has been collecting experimentally determined macromolecular structures. Currently, with over 200 000 entries, its structures come from macromolecular crystallography (MX), nuclear magnetic resonance (NMR) and, more recently, cryogenic electron microscopy (cryo-EM). The bulk of depositions derive from MX, but cryo-EM is on an accelerating trend (Callaway, 2020 ▸). Whatever the method, the experiment typically results in the determination of a model that is considered by the authors to (best) satisfy the observations. However, as with all scientific endeavour, experimental limitations can lead to unavoidable uncertainties and hence, despite the best efforts of experimentalists, the introduction of errors into the final model.

The 1990s saw a recognition of the need for error-detection or structure-validation tools, and a first generation of methods emerged. These were variously based on geometric and stereochemical properties [for example *PROCHECK* (Laskowski *et al.*, 1993 ▸) and *WHATIF* (Vriend, 1990 ▸)], consideration of statistics of favoured amino-acid environments (for example *VERIFY*3*D*; Lüthy *et al.*, 1992 ▸) or statistics of interatomic contacts [for example *ERRAT* (Colovos & Yeates, 1993 ▸) and *DACA* (Vriend & Sander, 1993 ▸)], or a combination of a C^β^–C^β^ (or C^α^–C^α^) potential and solvent-exposure statistics (*ProSA*; Sippl, 1993 ▸). More recent methods have further refined these concepts, for example *MolProbity* (Davis *et al.*, 2007 ▸), and have introduced measures of map–model scoring in programs such as *EMRinger* (Barad *et al.*, 2015 ▸), *Q-score* (Pintilie *et al.*, 2020 ▸) and *SMOC* (Joseph *et al.*, 2016 ▸). The very recent method *MEDIC* marries the geometric and the map-fitting approaches with special application to cryo-EM structures (Reggiano *et al.*, 2023 ▸). Of particular note, *Coot* (Casañal *et al.*, 2020 ▸) and *ISOLDE* (Croll, 2018 ▸) each now provide a sophisticated visualization of diverse structure-validation results at the interactive model-building stage of structure determination.

A key principle of structure validation is that the property used for validation should not be included in the target function during structure determination. Basic chemical features such as bond distances and angles therefore have limited validation utility since they are typically restrained to ideal values during structure determination, especially in recognition of the fact that many X-ray crystal structures are under-determined, with fewer observations than model parameters. The danger of failing to maintain a distinction between refined parameters and those used for validation is illustrated by the introduction of Ramachandran plot restraints, which have led to the emergence of structures that pass Ramachandran tests for outliers, but are shown by more sophisticated statistical analysis to exhibit highly skewed and implausible Ramachandran plot distributions (Afonine *et al.*, 2023 ▸). The ingenuity of researchers in finding novel, independent metrics such as *CaBLAM* (Prisant *et al.*, 2020 ▸) has proved important, but new validation metrics are still very valuable.

One particularly difficult class of errors to detect are sequence-register errors, in which the main chain may be broadly correct but residues are systematically assigned the identity of a residue a number of amino acids up or down in the sequence. Such errors are particularly easily introduced where the local resolution of the map is lower, and especially where more easily identifiable marker residues such as aromatic amino acids are absent. The fact that the backbone is often essentially correct in the region of the register error means that some conventional validation scores will struggle to detect a problem, although nonrotameric side chains and steric clashes may sometimes be helpful (Chojnowski, 2022 ▸, 2023 ▸). By representing residue probabilities at each C^α^ position in an input using a neural network classifier, the program *checkMySequence* has contributed significantly to the detection of register errors in both MX and cryo-EM structures (Chojnowski, 2022 ▸, 2023 ▸), but its dependence on map quality means that its sensitivity naturally declines at poorer resolution. The development of the DAQ score (Terashi *et al.*, 2022 ▸) and an associated database (Nakamura *et al.*, 2023 ▸) are further major contributions to structure validation, but this analysis is also dependent on map quality and resolution.

We have recently introduced new methods for protein structure validation (Sánchez Rodríguez *et al.*, 2022 ▸) based on the compatibility of a structure with the inter-residue distances and contacts predicted by methods such as *AlphaFold*2 (Jumper *et al.*, 2021 ▸). These were firstly a support vector machine (SVM) classifier which predicts whether a given residue is part of an error of any kind, and secondly a contact-map alignment procedure which detects whether the mapping between the observed residue contacts in a structure and the predicted contacts is optimal or whether an alternative alignment would score better: a sign of a possible register error. After specific steps to filter out potential false positives based on either a relative paucity of contacts for the potential error region or excessive structural divergence between the structure and the *AlphaFold*2 model, we found a combination of the SVM and contact-map alignment to be very effective in the detection of sequence-register errors. Notable features of our method are its map independence, making it insensitive to the resolution of the structure analysed, and its ability to use the contact-map alignment step to suggest the correction required to arrive at the correct sequence register.

Here, we present the results of validating all 3–5 Å resolution structures in the PDB, both cryo-EM and MX, using our new method. Even on applying stringent criteria, we identify thousands of putative register errors which we further validate by comparison with high-resolution crystal structures and with the orthogonal map-based validation tool *checkMySequence* (Chojnowski, 2022 ▸, 2023 ▸). Finally, automated correction of the likely errors results, in most cases, in improved local real-space correlation coefficients, adding further confidence that an error has been identified. While our method remains limited by factors that affect the prediction of residue distances and contacts by *AlphaFold*2, such as the depth of the multiple sequence alignment that can be constructed of the target sequence and homologues, and can be misled by rare fold-switching proteins, it is powerful and conceptually independent of structure-validation methods hitherto applied to PDB entries (Sali *et al.*, 2015 ▸; Kleywegt *et al.*, 2024 ▸). These results should therefore help to avoid much misguided research effort based on locally incorrect PDB structures (Gao *et al.*, 2023 ▸).

## 2. Materials and methods

### 2.1. Selection of protein models deposited in the PDB

The data set of protein structures used in this study was selected by first retrieving a list of all structures determined using cryo-EM or X-ray crystallography which were deposited in the PDB up to 5 April 2022 at resolutions between 3.0 and 5.0 Å. The resulting 19 310 PDB entries were then split into their constituent chains, leading to the creation of a data set of 203 533 protein chains. Chains solely formed by nucleotides or ligands were discarded. Additionally, chains with more than 1000 residues were also discarded as obtaining *AlphaFold*2 predictions would be intractable due to hardware limitations. The remaining set of 148 785 protein chains were then clustered according to their sequence identity: protein chains sharing 100% sequence identity were grouped together, resulting in the creation of a final data set of 30 731 clusters, each having an approximate average of five members. To ensure a match between the residue numbering observed in the deposited models and the numbering in the reference sequence deposited in the PDB used to obtain *AlphaFold*2 predictions, a pairwise sequence alignment was performed using the BLOSUM62 substitution matrix and the chains in these clusters were renumbered accordingly.

### 2.2. Prediction of contact maps derived from of inter-residue distances obtained using *AlphaFold*2

Predictions of inter-residue distances were obtained using *AlphaFold*2 for each of the clusters in the data set created as described in Section 2.1. Since all of the structures in each cluster have 100% sequence similarity, only one prediction was carried out for each individual cluster. For efficiency, the database search required as part of the *AlphaFold*2 run was carried out with *MMseqs*2 (Mirdita *et al.*, 2019 ▸) instead of the original *jackhmmer* search (Eddy, 2011 ▸). The original CASP14 model preset was then used and all parameters were left to their defaults. Using this setup, one predicted model was produced with *AlphaFold*2 for each of the clusters in the data set. The inter-residue distances for this predicted model were then taken. These predictions consist of the predicted probabilities that each residue pair in the structure is within a given distance bin. Contact predictions were derived from these inter-residue distance predictions by adding together all probabilities observed for the distance bins up to 8 Å for each residue pair. Finally, the top *L*/2 residue contacts scoring the highest probability values were taken to form the final predicted contact maps, where *L* denotes the sequence length of the protein chain.

All of these predictions were carried out on a computing grid where each node was equipped with a twin 16-core Intel Xeon Gold 5218 running at 2.3 GHz with 160 GB of memory and four NVIDIA Tesla V100 chips with 16 GB of video memory each.

### 2.3. Contact map alignment-based model validation

Model validation was carried out for each protein model present in the data set described in Section 2.1 using the contact-map alignment step of the *conkit-validate* pipeline (Sánchez Rodríguez *et al.*, 2022 ▸). In this pipeline, the contact map predicted by *AlphaFold*2 was aligned with the contact map derived from the residue distances observed in the deposited model using *map_align* (Ovchinnikov *et al.*, 2017 ▸). This tool introduces and extends gaps as required in order to obtain the optimal alignment between the two input contact maps, which is defined as the alignment where the maximum number of contacts match each other in both maps (contact maximum overlap; CMO). In cases where the maximum overlap between contacts was achieved using a sequence register different to that observed in the deposited model for at least five consecutive residues, the mismatch was flagged by *conkit-validate* as a possible sequence-register error and the sequence register that achieved the CMO was proposed as a possible fix for the predicted error. Additionally, this pipeline merged consecutive regions flagged as register errors if they were separated by three residues or fewer: it seemed unlikely that nearby predicted errors had independent causes and so counting them once seemed fairer. As a final step, errors predicted with this pipeline were filtered using three criteria established in a previous study (Sánchez Rodríguez *et al.*, 2022 ▸). These criteria were designed in order to discard predicted errors detected in parts of the model where the contact-map misalignment could be caused by reasons other than a register error. The first filter discarded predicted errors where either the mean or the median number of predicted contacts per residue observed across the affected residue range was less than two. The second filter eliminated predicted errors affecting residues where the average *AlphaFold*2 pLDDT was <65, thereby avoiding calling an error based on lower-confidence predictions. Finally, a structural alignment between the deposited model in which a register error was putatively detected and the model predicted by *AlphaFold*2 was performed using *GESAMT* (Krissinel, 2012 ▸). The *GESAMTQ*-score was then calculated for the range of residues affected by the possible register error. Where the *GESAMTQ*-score for the residues of the putative register error was below 0.5, the putative error was again discarded. Filtering out instances where the deposited model and the predicted model were very different addressed cases where the *AlphaFold*2 predictions could be inaccurate or the predicted model was modelled in a different conformation to that deposited.

### 2.4. Selection of a high-resolution crystal structure for cross-validation

In order to obtain a set of high-resolution structures that could be used to cross-validate the predicted register errors detected in this study, an advanced search was carried out in the PDB to find entries that share 100% sequence identity with each of the clusters in the data set described in Section 2.1. To restrict analysis to higher-resolution, generally more confident structures, the results of this search were then filtered so that only structures determined at least at 2.5 Å resolution and using X-ray crystallography were left. In cases where multiple entries meeting these criteria were found, the structure determined at the highest resolution was taken.

### 2.5. Modification of sequence register in models with predicted errors

For the deposited models where a possible sequence-register error was detected, a model with the alternative sequence register predicted to be correct was created as follows.

Firstly, for each error detected in the structure, a buffer was added at each side of the predicted error to account for cases where the register error was preceded or followed by a modelling error. The length of this buffer was proportional to the predicted residue-shift error, and was calculated as
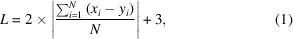
where *L* is the length of the buffer, *N* is the number of residues affected by the predicted error, *x* is the residue number observed in the deposited structure and *y* is the number of a corresponding residue predicted to be correct.

In those instances where the buffer regions of two contiguous errors shared any number of residues, the errors were joined and considered as a single error for the purpose of creating a model with the alternative sequence register, but they stayed as separate errors for the rest of the analysis.

Next, a C^α^-based sequence-independent structural alignment between the deposited model and the model predicted by *AlphaFold*2 was carried out using *GESAMT* (Krissinel, 2012 ▸). Those residues predicted to be part of a sequence-register error were then removed from the deposited model, together with their buffers, and replaced with the equivalent set of residues present in the superposed predicted model, which were modelled with the register predicted to be correct. *Coot* (Emsley *et al.*, 2010 ▸) was then used to perform real-space refinement on each pair of residues where the original deposited model was adjacent to the fragment originating from the predicted model. Further refinement and calculation of quality metrics was then performed on both the deposited model and the newly created model with the alternative sequence register. This was carried out in a different manner depending on the method used to determine the deposited model.

In cases where the structure was originally determined using cryo-EM, *REFMAC*5 (Murshudov *et al.*, 2011 ▸) was used to perform 20 cycles of jelly-body refinement both on the original model and the alternative model. The local correlation coefficient between the map and the modified set of residues in both the original and the alternative model was then calculated using *phenix.map_model_cc* from the *Phenix* suite (Liebschner *et al.*, 2019 ▸).

For MX cases, after correcting the predicted sequence-register errors, all of the chains present in the asymmetric unit, regardless of whether an error was detected in them or not, were used as input to perform 20 cycles of restrained refinement using *REFMAC*5 to help fit the modified region to the density. To allow like-for-like comparisons, 20 cycles of restrained refinement using default restraints and automatic weights were also run on the deposited structure. *density-fitness* (Hekkelman, 2023 ▸) from the *PDB-REDO* suite of programs (Joosten *et al.*, 2012 ▸) was used to calculate a per-residue real-space correlation coefficient (RSCC). By iterating through the residues of interest, a mean local RSCC was calculated for the modified and unmodified structures in the region of the predicted register error. To visualize the output MTZ files in *Chimera*, the MTZ files were converted to MRC files using *Coot*.

### 2.6. Alternative validation methods

#### 2.6.1. checkMySequence

The map–model compatibility tool *checkMySequence*was applied to all cryo-EM or crystal structures in the set using the procedures described previously (Chojnowski, 2022 ▸, 2023 ▸).

#### 2.6.2. Geometry

Unusual geometric features (Ramachandran outliers, side-chain rotamer outliers and C^β^ distortions) were detected with *MolProbity* (Prisant *et al.*, 2020 ▸; Davis *et al.*, 2007 ▸).

#### 2.6.3. DAQ scoring

To further assess the register errors in the cryo-EM structures, the average DAQ score across the residues corresponding to each predicted error was mined from the DAQ score database (Terashi *et al.*, 2022 ▸). These were compared with the delta *phenix.map_model_cc* score between the original and alternative models.

## 3. Results and discussion

### 3.1. One in six models determined at 3–5 Å resolution deposited in the PDB contains a putative register error

**Figure 1:**
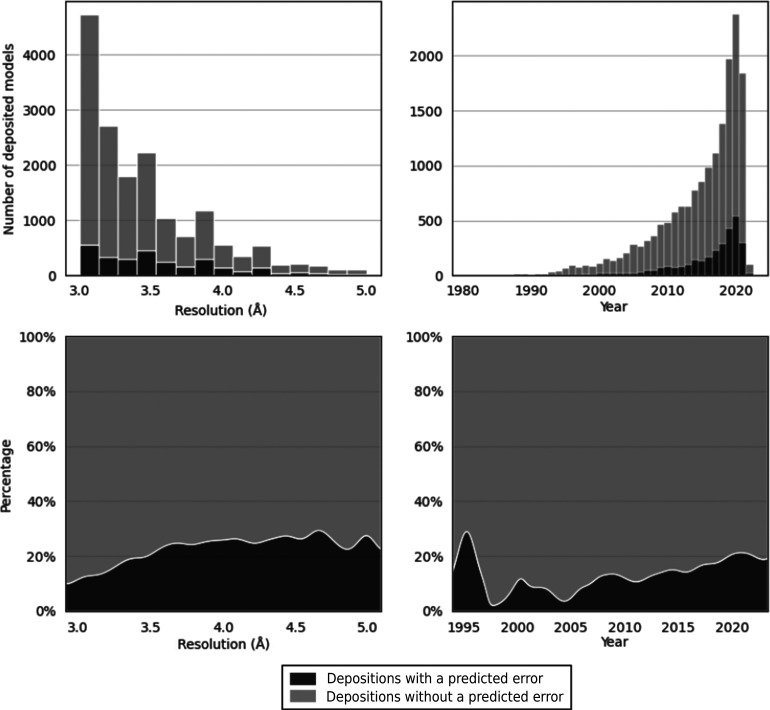
Distribution of models deposited in the PDB where a sequence-register error was predicted across different resolution bins (left column) or year of deposition (right column). Plots in the top row depict the stacked count of deposited models without a predicted error (light grey) and models where an error was predicted (dark grey). Plots in the bottom row show the percentage of deposited structures where an error was predicted. Depositions before 1995 have been omitted for clarity because the number of entries is small (only 201 in total from 1981 to 1995). Note that sequence-redundancy removal between different PDB entries has not been applied here: some entries represented here will contain the same error.

Processing of PDB entries at between 3 and 5 Å resolution as outlined in Section 2 produced a set of 16 662 structures. These were processed using the *conkit-validate* pipeline, with the purpose of detecting possible register errors. During this analysis, a total of 12 674 possible register errors were found distributed among 2954 entries (17%). When taking into consideration the fact that some sequences are represented several times in the data set in the form of homomeric structures or the same protein in different PDB entries, the total number of unique predicted register errors was 4606. Analysis of the distribution of PDB depositions with at least one predicted error across different resolution bins and deposition years was carried out (Fig. 1 ▸). Unsurprisingly, predicted errors are more common in lower-resolution entries: around 10% of entries at 3.0 Å resolution contain a predicted error, increasing to around 20% between 3.7 and 5.0 Å resolution. Perhaps less expected is the historical trend towards a larger number of predicted errors recently: only 8% of entries deposited between 1995 and 2000 contain a predicted error, increasing to almost 20% in recent years. However, this presumably reflects the increasing presence of cryo-EM in structure determination more recently and the encouragement that its experimentally measured maps offer to lower-resolution structural analysis (see below).

**Figure 2:**
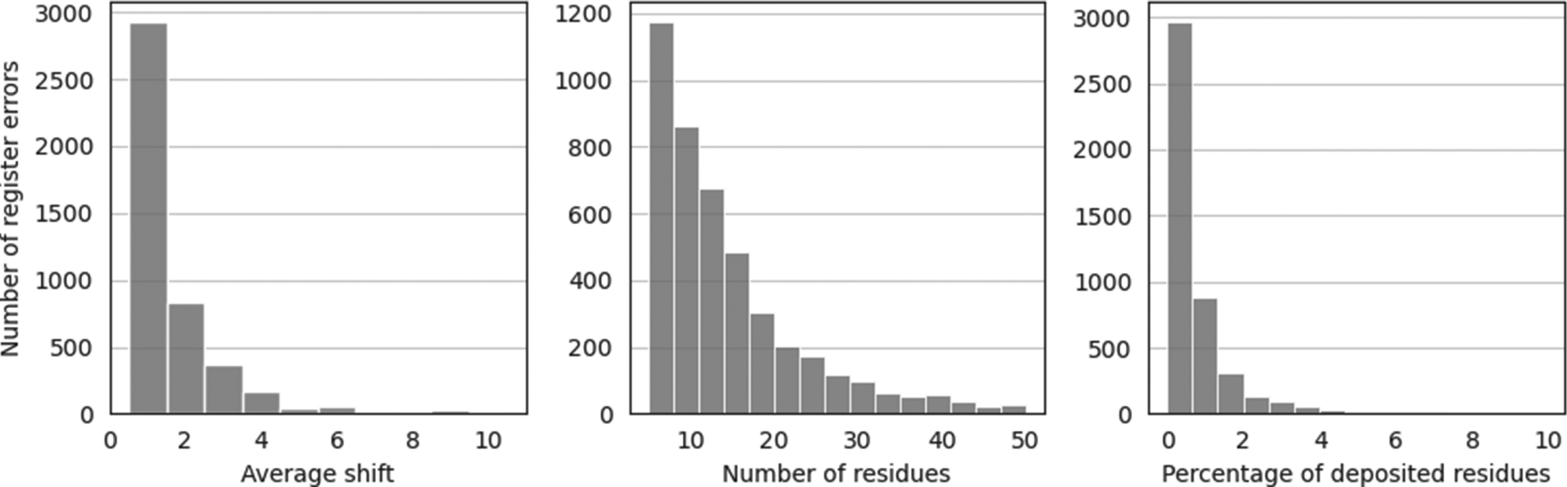
Number of predicted sequence-register errors plotted by the predicted residue shift (left), by the number of affected residues (middle) and by the fraction that the affected residues represent compared with the overall structure (right). Errors consisting of a shift of more than ten residues, affecting more than 50 residues or more than 10% of the residues in the structure have been omitted for clarity (127, 248 and 27 errors, respectively). Note that where two errors were separated by three residues or fewer they were combined (see Section 2), meaning that where the two errors involved shifts of different numbers of residues, the average shift for a predicted register error may be non-integral. Note that the numbers here are after sequence-redundancy removal and represent unique errors within and between PDB entries.

Further characterization of the predicted errors was carried out by studying the sequence shift predicted to be required to correct the error, the number of residues affected by the possible error and the fraction of residues present in the overall structure that were found to be part of the error (Fig. 2 ▸). Most of the predicted errors are small, with two-thirds affecting 15 residues or fewer. The shifts required to correct predicted errors are also small: two-thirds involve a single residue.

### 3.2. Errors are more common among PDB depositions determined by cryo-EM than by MX

**Figure 3:**
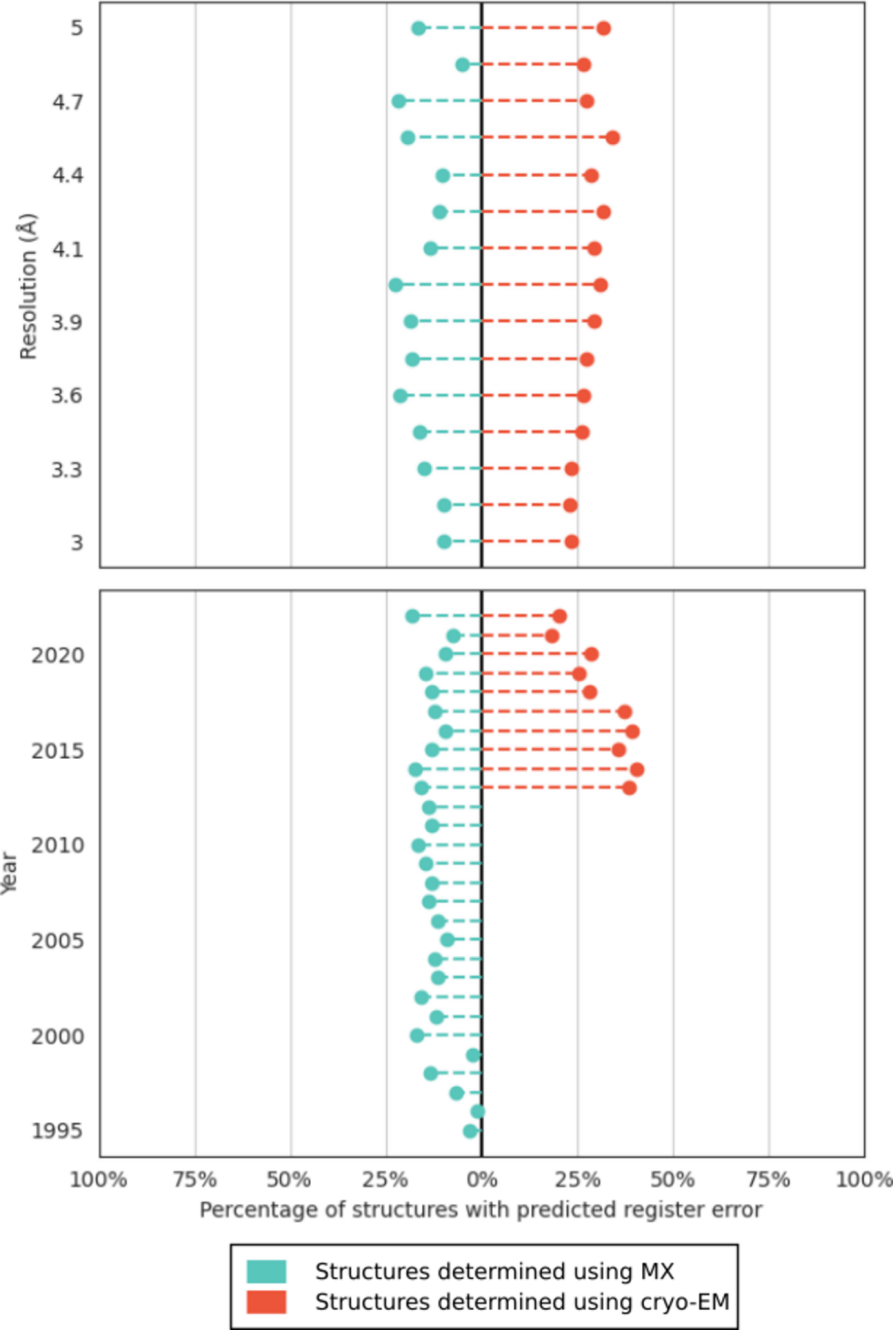
Proportion of structures containing a register error across different resolutions and years. Ratios for structures determined using MX are coloured blue, while those determined using cryo-EM are in red. Data for MX structures deposited before 1995 and for cryo-EM structures deposited before 2013 are small in number and have been omitted for clarity (136 and 28 entries, respectively).

A comparison of the distribution of predicted errors found in structures solved using cryo-EM or MX was carried out. Interestingly, a lower ratio of structures containing errors was observed for MX, regardless of the year of submission or the resolution bin. In contrast to the MX error incidence, which was broadly stable over time, the proportion of cryo-EM structures which contain a predicted error was observed to decrease over recent years, with values approaching those observed among MX structures (Fig. 3 ▸).

Breaking down the incidence of cryo-EM and MX predicted errors into resolution bins (Supplementary Fig. S1) suggests that the recent reduction in the incidence of predicted registered errors for cryo-EM structures seems to be centred principally on the 3.5–4.5 Å resolution range.

### 3.3. High-resolution crystal structures support the existence of many predicted errors

We used the same error-detection method to analyse high-resolution counterparts of crystal structures from our low-resolution set. We searched for structures with 100% sequence identity that were solved using MX at a resolution of at least 2.5 Å. In total we found 147 structures that met these criteria: our proposal is that where the later (in 79% of cases), higher-resolution structure does not contain the predicted error found in its lower-resolution predecessor, then the correction of an error with the benefit of better data is the likely explanation. In many cases both high- and low-resolution counterpart structures will have been present in the training set of *AlphaFold*2, but we expect that contacts and distances inferred from evolutionary covariance information should nevertheless be compatible uniquely with the correct register. These 147 structures covered 403 unique errors representing, since there were 4606 unique errors in total, approximately 10% of the unique errors (*i.e.* after sequence-redundancy removal) that we found in the original set of depositions. This small set of errors is representative of the full set of errors found in this study in terms of the distribution of register error size and shift (Supplementary Fig. S2).

**Figure 4:**
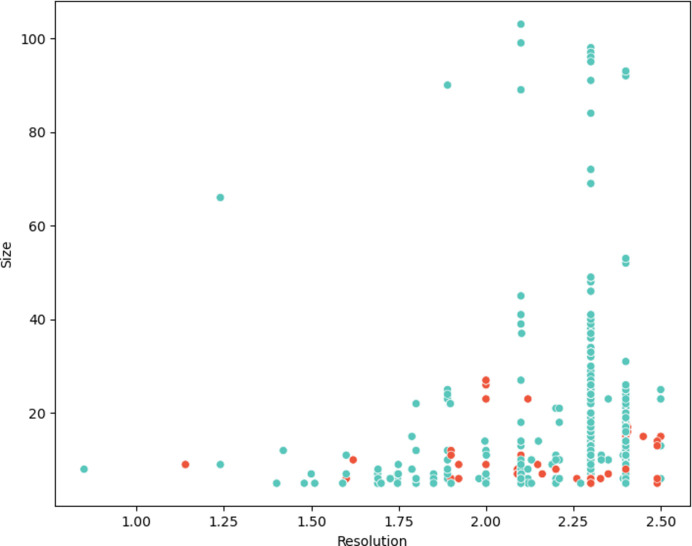
Comparison of 403 predicted unique errors with 147 high-resolution MX structures. Each predicted unique error is plotted according to the resolution of the structure in question and the number of residues affected by the predicted error. Blue points indicate cases where no error is detected in the high-resolution crystal structure and red points indicate where this is not the case.

After repeating the exercise on the set of 147 structures, no register error was predicted using our approach for 115 of these high-resolution depositions, supporting (since these structures encompassed 350 predicted errors) 87% of these unique predicted errors (Fig. 4 ▸).

Supplementary Fig. S3 shows a typical case where the electron density unambiguously supports the sequence register in the high-resolution (1.42 Å) structure (of Ragulator complex protein LAMTOR4; PDB entry 6b9x, chain *D*, residues 24–36), thereby supporting the prediction of a register error in the lower-resolution (3.01 Å) structure (PDB entry 5yk3, chain *I*).

Cases where a predicted error is called in the lower-resolution structure but also in the higher-resolution structure are more surprising where the quoted resolution would generally be considered sufficient to allow unambiguous sequence tracing. The three highest-resolution examples were examined in more detail.

The first was a predicted one-residue register-shift error spanning eight residues from positions 63 to 70 in the 3 Å resolution MX structure of an *Escherichia coli* FimH complex (PDB entry 4xob, chain *C*). The 1.14 Å resolution MX structure of the same protein (PDB entry 4xo9, chain *A*) was also predicted to have a register error in the same region and had a similar local structure. The *AlphaFold*2 model had a different register in the region, which was the source of the predicted error in both MX structures, but, unexpectedly, a further high-resolution 1.0 Å resolution structure of (one domain of) FimH (PDB entry 4x5p, chain *A*) shared the *AlphaFold*2 register. The electron density for each high-resolution structure unambiguously supports the atomic interpretation [Supplementary Fig. S4(*a*)], suggesting that the region is genuinely structurally ambivalent. A possible explanation for the difference lies in the observation that the stretch immediately prior to the predicted register error differs in the structure, forming either an uninterrupted β-strand (PDB entry 4x5p) or a β-bulge (Richardson *et al.*, 1978 ▸; PDB entry 4xo9); indeed, this difference triggers the downstream register difference. Crucially, however, this surface-exposed stretch is at the crystal lattice interface in each of the structures, and the lattice differs since the crystals are in different space groups [Supplementary Fig. S4(*b*)]. In particular, hydrogen bonds made by the side chain of Arg60 in each form may help to drive the local structural difference [Supplementary Fig. S4(*c*)]. Fold-switching proteins with more dramatic differences between alternative biologically relevant structures are considered below.

The second example involves structures of tick anti­coagulant peptide at both high resolution (1.62 Å; PDB entry 1dod; chain *A*) and low resolution (3.0 Å; PDB entry 1kig; chain *I*). They share a one-residue register shift with respect to the *AlphaFold*2 model over residues 19–28. Although, unfortunately, no diffraction data are available, the crystal structures also share the same register with two NMR structures of the same protein (PDB entries 1tcp and 1tap), suggesting that here the *AlphaFold*2 model, and hence the error prediction, is either straightforwardly wrong or captures an alternative legitimate conformation. Notably, this protein contains three disulfide bonds and some of the literature has suggested that these may lead to difficulties for *AlphaFold*2 (Thornton *et al.*, 2021 ▸; Wehrspan *et al.*, 2022 ▸). It is also worth noting that the *AlphaFold*2 model can be fitted to the crystal structures only rather poorly, with a 2.89–3.03 Å r.m.s.d. on 59 C^α^ atoms.

The third example involves structures of *Methylophilus methylotrophus* flavoprotein at high resolution (1.60 Å; PDB entry 1o97; chain *A*) and low resolution (3.1 Å; PDB entry 1o96; chain *Z*). They share a one-residue register shift with respect to the *AlphaFold*2 model over residues 196–201. Immediately prior to this region they each have a number of residues, three or six, that are not traced in the final structure: this relates to the lower local quality of the electron density in the domain-linker region. Although not allowing immediate and unambiguous interpretation, the electron density calculated using the higher-resolution structure seemed to be more consistent with the *AlphaFold*2-preferred register (not shown). Indeed, the register matching the *AlphaFold*2 model is seen in structures deposited six years later by the same group, for example PDB entry 3cls at 1.65 Å resolution.

### 3.4. Local correlation values improve when predicted errors are corrected

**Figure 5:**
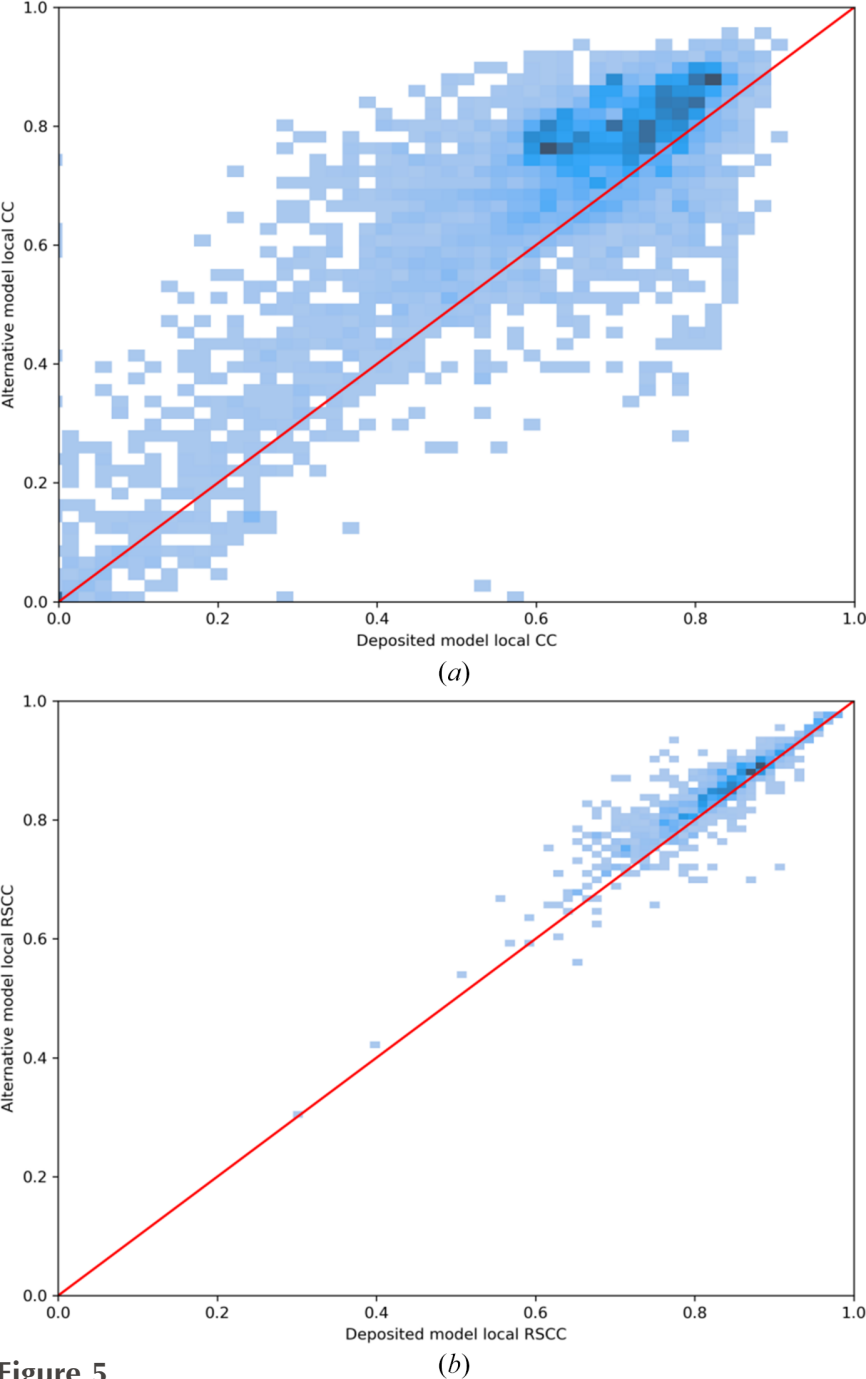
Density plots illustrate that local CC values improve for most (*a*) cryo-EM (*Phenix* map–model correlation coefficient) and (*b*) MX (*density-fitness* real-space correlation coefficient) cases after the correction of predicted errors.

Deposited models in which a sequence-register error was predicted were corrected as described in Section 2.5. Then, either the *density-fitness* real-space correlation coefficient (RSCC; MX cases) or the *Phenix* map–model correlation coefficient (CC; cryo-EM cases) was calculated for the stretch of residues that were modified and compared with the coefficient achieved with the set of residues present in the original deposition. Comparison of these local correlation values revealed that the CC improved in 4522 of the 5599 cryo-EM cases (80%) and the RSCC improved in 3266 of the 4059 MX cases cases (80%) (Fig. 5 ▸).

### 3.5. Comparison with other validation tools

#### 3.5.1. New-generation map-based validation with *checkMySequence*

We used *checkMySequence* (*cMS*) to look for possible register errors within the structures in our data set. *cMS* is based on map–model compatibility and hence is a completely independent approach. Since *cMS* is based on comparison of the model with the experimental data, only those models that had experimental data deposited could be analysed. Of the 16 662 PDB entries that we analysed, 12 879 met this requirement. Furthermore, due to the underlying methods used within* cMS*, only register errors spanning at least ten residues will be flagged. This means that out of the 4606 ‘unique’ errors that we found in the data set, only 1556 could be potentially found by *cMS* (33%).

**Figure 6:**
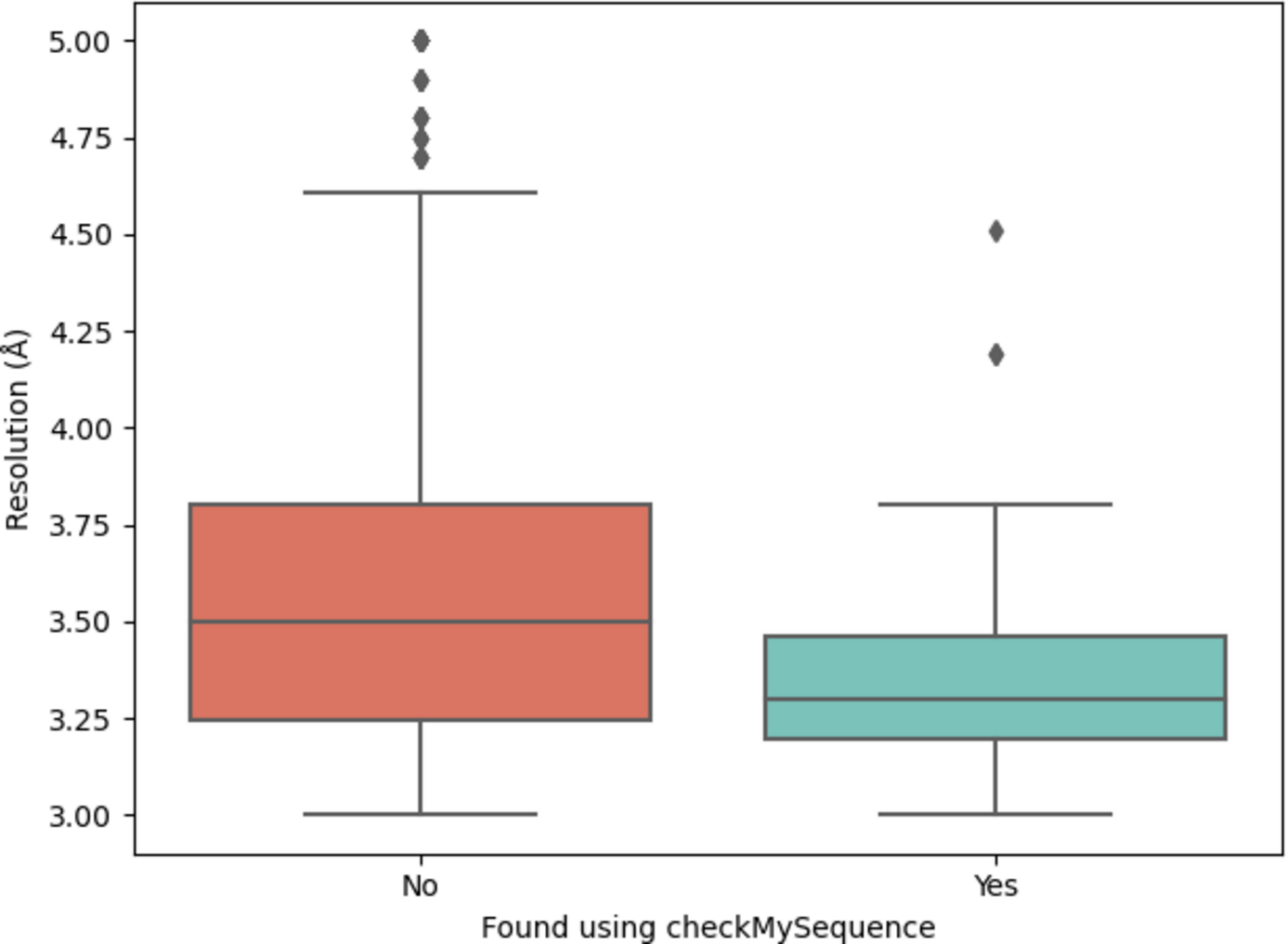
For those deposited structures where a possible sequence register was predicted by *conkit-validate* and experimental data were available, comparison of the distribution of the resolutions at which they were solved and whether a possible register error was also predicted by *checkMy­Sequence*.

A total of 672 errors were predicted by *cMS* among the 12 879 entries in the data set that contained experimental data. Of these, only 90 were part of the set of 1556 errors also predicted by *conkit-validate*. A comparison of the resolution distributions of the deposited structures where an error was or was not found using *cMS* is shown in Fig. 6 ▸. The distribution of predicted errors extending to lower-resolution cases in the set found by *conkit-validate* alone illustrates the valuable resolution independence of the program.

Additionally, analysis of the structures where a sequence-register error was flagged by *cMS* but not by *conkit-validate* revealed that 70% had been predicted to contain the same error by *conkit-validate*, but these errors were discarded by the filters described in Section 2.3 and established in a previous study (Sánchez Rodríguez *et al.*, 2022 ▸). This suggests that there is room for improvement in the filtering of results that we previously found necessary to reduce the incidence of false-positive results (Sánchez Rodríguez *et al.*, 2022 ▸), *i.e.* it has removed some true positives that were also discovered by *cMS*.

#### 3.5.2. Model geometry

**Figure 7:**
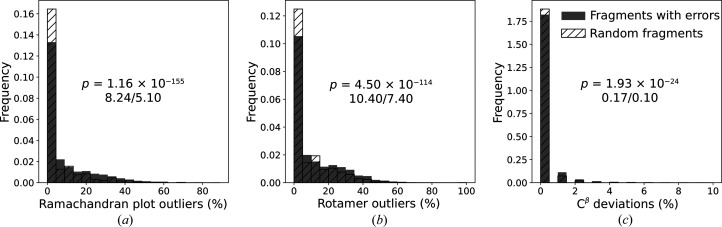
Comparison of distributions of (*a*) Ramachandran plot outliers, (*b*) rotamer outliers and (*c*) C^β^ deviations detected using *MolProbity* in model fragments with plausible register shifts (grey bars) and random fragments (hatched bars). The *p*-values shown on the plots correspond to a two-sample *t*-test with a hypothesis that the expectation value for random fragments is smaller. The numbers below the *p*-values are mean fractions of outliers for fragments with errors and random fragments, respectively.

It has been observed that register shifts may result in local concentrations of model-geometry violations (Chojnowski, 2022 ▸, 2023 ▸), so we evaluated the fractions of various geometry errors identified using *MolProbity* (Ramachandran plot outliers, rotamer outliers and C^β^ deviations) in model fragments with identified register shifts and random fragments of comparable lengths selected from the same models. We observed that the fraction of errors is indeed larger in regions with putative errors, but the difference is relatively small for all of the validation criteria tested (Fig. 7 ▸). This clearly supports our previous observation that register shifts may result in a locally increased number of validation outliers. However, it is clear that many regions with confident putative register errors that are identifiable with our new method lack geometric issues that alone would enable their identification.

#### 3.5.3. The composite cryo-EM validation measure DAQ score

**Figure 8:**
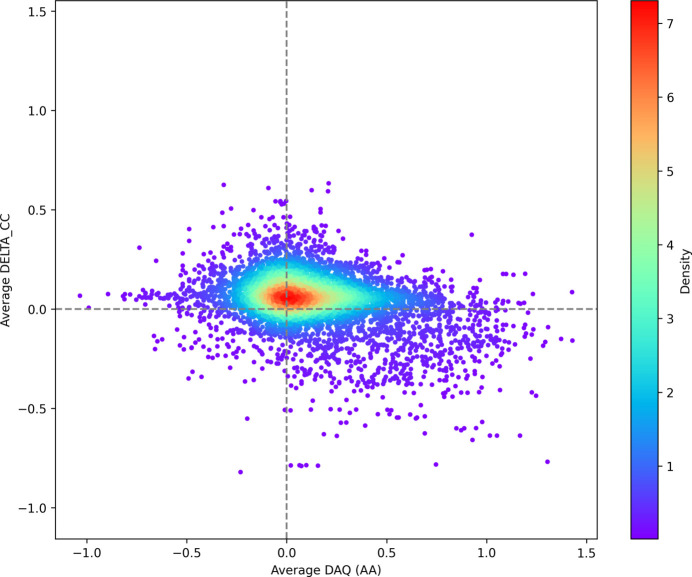
A scatter diagram showing average delta in *Phenix* map–model CC before and after correction versus the average DAQ score over the predicted error. The points in the plot are coloured by density, where blue represents a low density of points and red represents a high density of points.

We further assessed the predicted register errors for the cryo-EM targets by comparing the average ΔCC score between the original and the alternative models against the average DAQ score over the predicted register error (Fig. 8 ▸). Where the DAQ score indicated a potential error [average DAQ(AA) < 0] we observed that ΔCC improved in 1699 of the 2136 cases (79.5%). Where no error was indicated by the DAQ score [average DAQ(AA) > 0] we observed that ΔCC improved in 2525 of the 3758 cases (67.2%). It should be noted that in a large number of these cases the average DAQ score was very close to zero, indicating that the map lacked distinctive map features that would allow the DAQ neural network to make a decisive call. This helps to highlight the importance of our map-independent method when looking for potential errors.

### 3.6. Selected examples

#### 3.6.1. Cryo-EM examples

**Figure 9:**
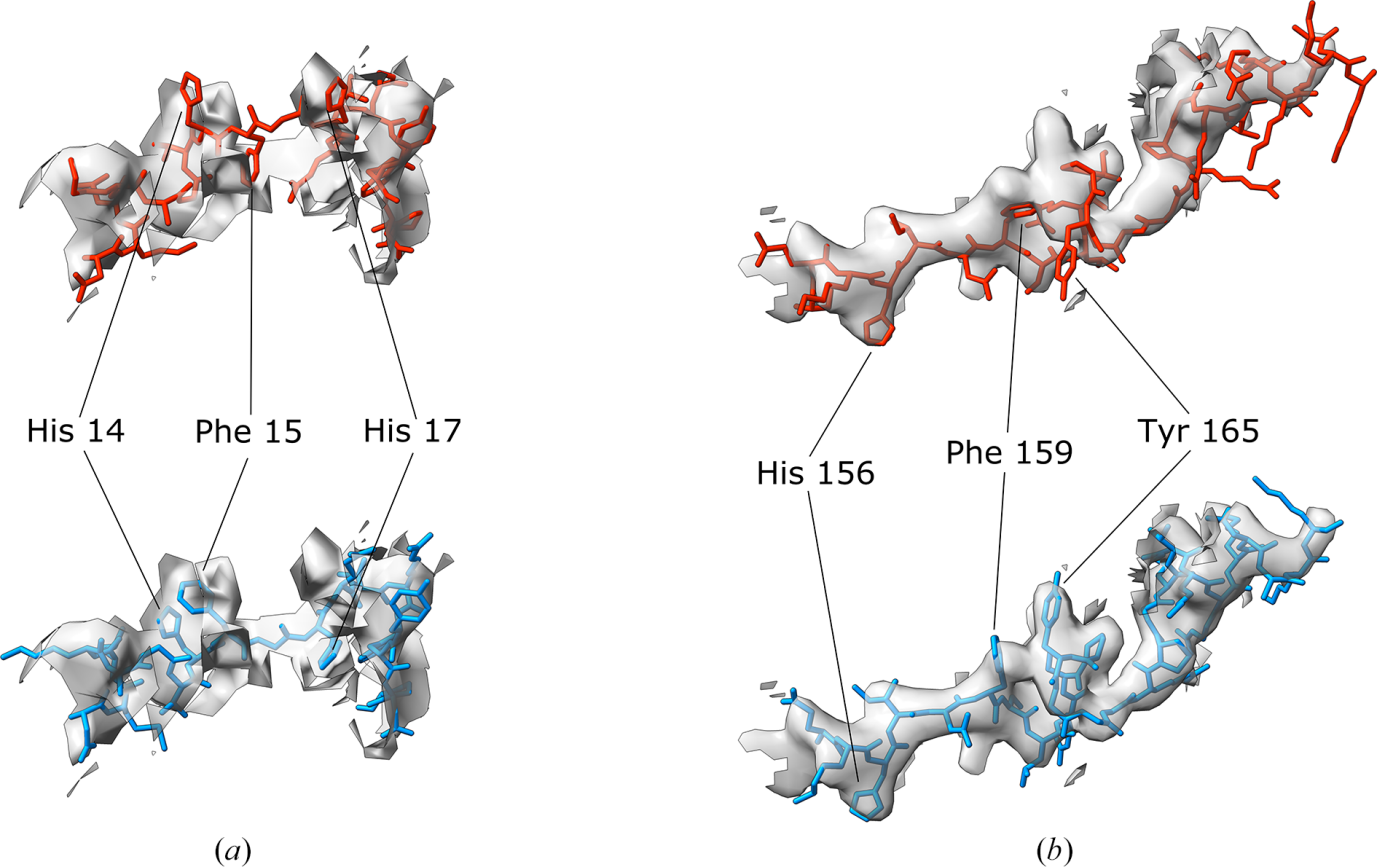
Detailed views of portions of deposited cryo-EM structures in which a possible sequence-register error was detected using *conkit-validate*. (*a*) The error corresponds to PDB entry 3j9z chain SB residues 7–24, and residues 14, 15 and 17 have been highlighted. (*b*) The error corresponds to PDB entry 6gz3 chain BJ residues 153–180, and residues 156, 159 and 165 have been highlighted. In each case, a mask of 2.5 Å around the model was applied; the original deposition is coloured red, with the structure corrected with the sequence register suggested by *conkit-validate* in blue. The density map for the deposited structure is represented as a transparent grey surface with the contour level set to 0.035 in (*a*) and 2.2 in (*b*).

Fig. 9 ▸ illustrates two representative predicted errors discovered by *conkit-validate*. The first, also flagged by *cMS* [Fig. 9 ▸(*a*)], indicates a predicted 18-residue error in chain SB (note that we use author chain IDs throughout) of the *E. coli* 30S ribosomal protein S2 in a ribosome structure, PDB entry 3j9z, determined to a reported resolution of 3.6 Å. The second, found solely by *conkit-validate* [Fig. 9 ▸(*b*)], illustrates a predicted 28-residue error in chain BJ of the rabbit ribosome structure, PDB entry 6gz3, also at 3.6 Å resolution. Notably, the regions in question, when analysed by *CryoNet* (Dai *et al.*, 2023 ▸), appear to have a local map resolution that is significantly worse than the overall quoted values and certainly worse than 4 Å (Supplementary Fig. S5). In each case, bulky, aromatic side chains provide visual confirmation that the new register is correct. Indeed, the local map–model CC for the region in question improves dramatically after correction of the error as suggested by *conkit-validate*: for PDB entry 3j9z it improves from 0.29 to 0.71, while the values before and after correction for PDB entry 6gz3 are 0.51 and 0.82, respectively.

#### 3.6.2. MX examples

**Figure 10:**
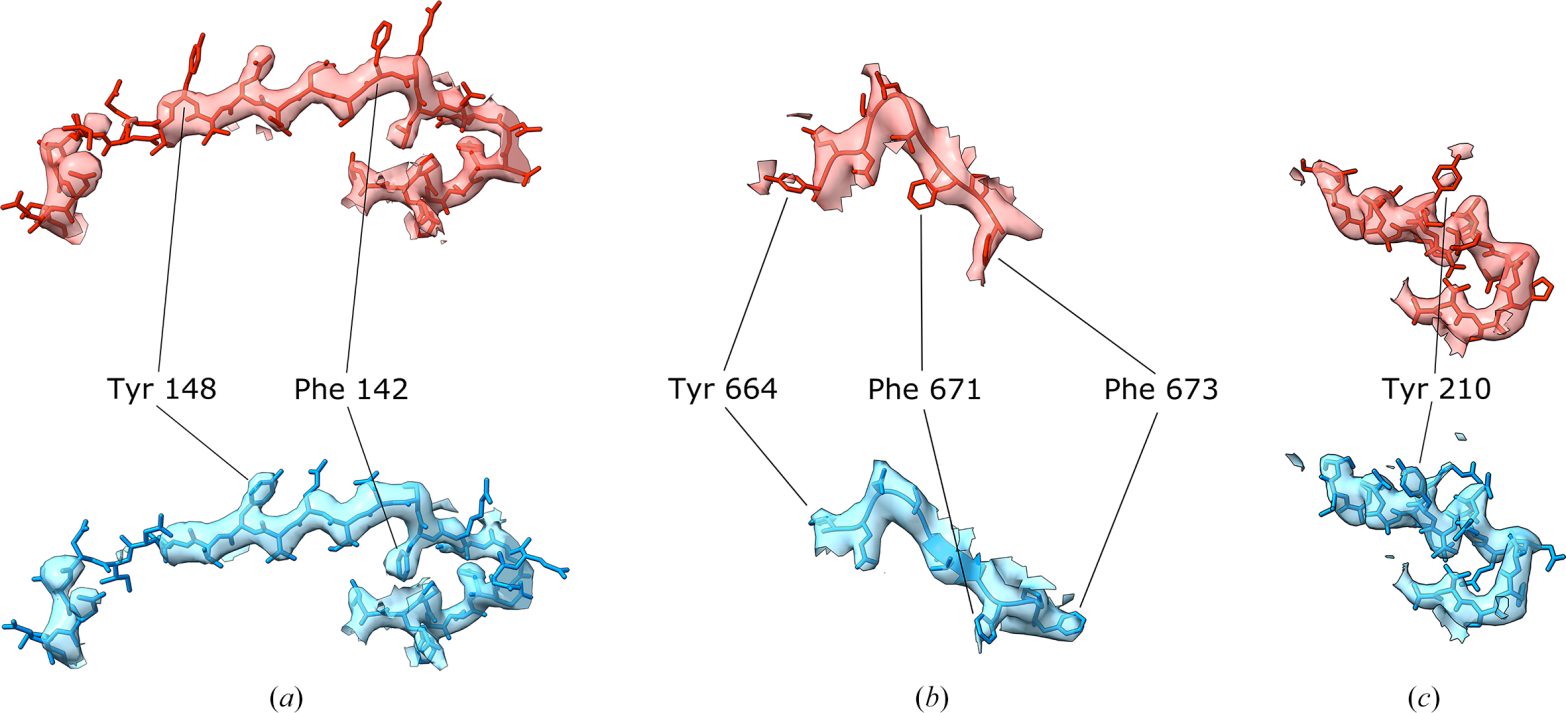
Detailed views of portions of deposited MX structures in which a possible sequence-register error was detected using *conkit-validate*. (*a*) The error corresponds to PDB entry 2yks chain *A* residues 130–157, and residues 142 and 148 have been highlighted. (*b*) The error corresponds to PDB entry 5k8d chain *A* residues 664–673, and residues 664, 671 and 673 have been highlighted. (*c*) The error corresponds to PDB entry 6yl7 chain *A* residues 199–219, and residue 210 has been highlighted. In each case, a mask of 2.4 Å around the model was applied. The maps are 2*F*_o_ − *F*_c_ maps coloured at the same contour level, with the original deposition in red and the structure corrected with the sequence register suggested by *conkit-validate* in blue. The contour levels are 0.207, 0.154 and 0.24 in (*a*), (*b*) and (*c*), respectively.

Fig. 10 ▸ shows representative examples of predicted register errors in crystal structures before and after correction. The predicted errors are found in a bacterial ion channel determined to 3.3 Å resolution [Fig. 10 ▸(*a*)], a human coagulation factor complex at 4.19 Å resolution [Fig. 10 ▸(*b*)] and a bacterial carbonic anhydrase at 3.17 Å resolution [Fig. 10 ▸(*c*)]. The first two were also detected by *cMS*, while the last was not. The case in Fig. 10 ▸(*a*) is the same error as highlighted in a cryo-EM structure in our previous paper (Sánchez Rodríguez *et al.*, 2022 ▸), illustrating the map-agnostic nature of our method.

#### 3.6.3. Fold-switching

**Figure 11:**
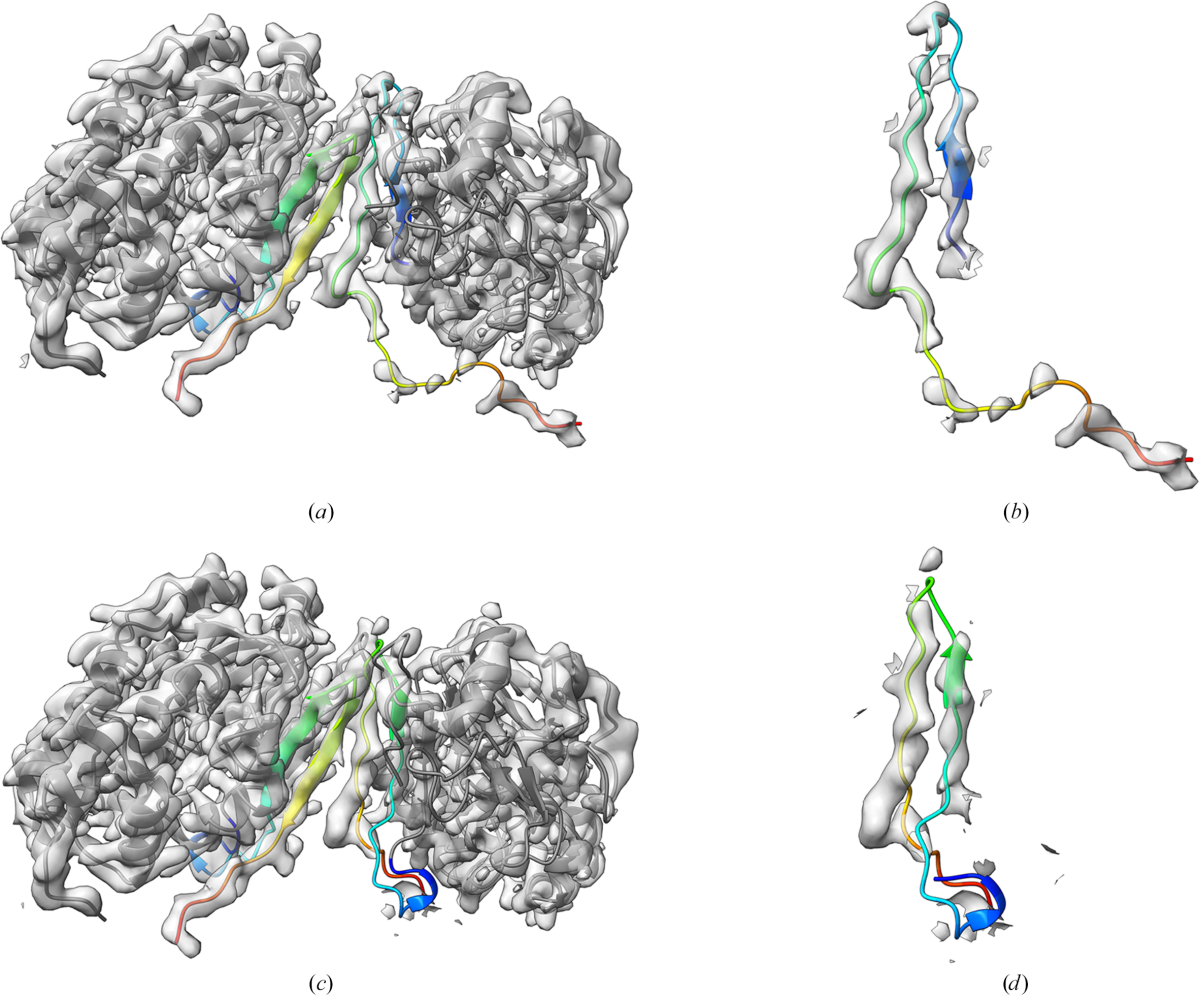
Detailed view of the section of the deposited model where *conkit-validate* erroneously identified a potential sequence-register error in human calcineurin (PDB entry 5c1v). (*a*) The deposited structure is shown in grey with the fold-switching region (residues 309–340) shown in a rainbow spectrum, where blue indicates its N-terminal part and red indicates the C-terminus. The density map for the deposited structure is represented as a transparent grey surface and the contour level was set to 0.25. (*b*) A close-up of the fold-switching region in chain *B* of the deposited structure and the associated density. (*c*) The automated attempt at fixing the potential sequence-register error identified by *conkit-validate*. (*d*) A close-up of the fold-switching region in chain *B* of the attempted fix.

Our method depends on spotting mismatches between a deposited structure and the structure implied by deep-learning-based prediction of inter-residue distances and contacts. It will therefore potentially struggle with the small proportion of proteins, known as fold-switchers, which have alternative, distinctly different, but equally biologically valid folds. We find one such example in our set, human calcineurin (PDB entry 5c1v, determined by X-ray crystallography to 3.35 Å resolution), which undergoes a significant rearrangement of C-terminal β-strands as a result of reversible *cis*–*trans* isomerization of a proline residue (Guasch *et al.*, 2015 ▸). In chain *A* of the structure *trans*-Pro309 adopted the previously universal structure, whereas *cis*-Pro309 in chain *B* led to a novel conformation in the C-terminal region and, in particular, a different sequence register of two β-strands compared with chain *A* (Fig. 11 ▸). Electron density provides good support for the novel conformation [Fig. 11 ▸(*b*)], yet residues 288–340 are flagged as a potential error by *conkit-validate*. The attempted ‘correction’ of register leads to a confirmation resembling that of chain *A* which has a poorer fit to the density [Fig. 11 ▸(*d*)], demonstrating that this case is a false-positive error prediction.

## 4. Conclusions

We have presented a PDB-wide analysis of deposited structures determined to between 3 and 5 Å resolution using our new validation protocol. It is based on deep-learning-derived predictions of inter-residue distances and contacts, but the availability of an associated 3D structure prediction provides extra confidence by enabling the filtering out of some false positives (Sánchez Rodríguez *et al.*, 2022 ▸). Potential errors are flagged as disparities between the observed residue distances and contacts and those predicted by the latest generation of deep-learning-based tools, most notably *AlphaFold*2 (Jumper *et al.*, 2021 ▸). Although general in its ability to detect errors, our method is particularly effective when applied, as here, to the detection of register errors (Sánchez Rodríguez *et al.*, 2022 ▸). Importantly, our method is orthogonal to current validation metrics, providing a valuable further tool for the detection of errors in protein structures. Also notably, it is independent of the experimental map, be it cryo-EM or crystallographic, and hence its performance is unaffected by (local) resolution.

We found that around one in six PDB depositions determined to 3–5 Å resolution contained a predicted register error, although this amounts to only 2.3% of residues. Given the benchmarking previously carried out, the implementation of filters to catch false positives and the observable improvements in map–model compatibility after correction (Fig. 5 ▸), we are confident that these represent high-confidence predictions. The large numbers involved allow trends to be analysed: unsurprisingly, entries in the higher-resolution part of the studied range are less likely to contain a predicted error than lower-resolution depositions. We find that cryo-EM structures are more likely to contain a predicted error than crystallo­graphic structures at the same quoted resolution, but it must be borne in mind that cryo-EM maps, especially, often contain regions in which the resolution is much poorer than the headline value. In addition, the gap in error frequency between the two methods seems to be narrowing recently.

While powerful, our method has limitations. Most obviously, it depends on the quality of the distance and contact predictions available. In most cases, the quality is very high, as illustrated by the superb accuracy achieved by methods such as *AlphaFold*2 (Jumper *et al.*, 2021 ▸; Pereira *et al.*, 2021 ▸; Simpkin *et al.*, 2023 ▸). However, recent analysis demonstrates that contemporary modelling methods of different kinds still consistently struggle with targets that are singletons or for which very few homologues can be found in databases. In addition, small size and high α-helical content may be additional aggravating factors (Simpkin *et al.*, 2023 ▸). In such cases, where accurate modelling is not possible, the corresponding distance and contact predictions will also be lower quality and hence, potentially, not suitable for use in validation. In this regard, we have also presented an example in which the presence of disulfide bonds in the target may have resulted in an unusually low quality *AlphaFold*2 prediction. As we have also shown, our method can also misbehave in rare cases of fold-switching proteins, flagging a potential error in the part of the structure that has two biologically valid structures. A similar situation will arise in very unusual cases of homologous proteins that adopt entirely different structures (Schierholz *et al.*, 2024 ▸). More positively, such a misprediction in regions of confident structure building could potentially be interpreted as a sign of a potentially interesting fold-switching region or a different kind of biologically relevant structural ambivalence. It may also be that future work looking at *AlphaFold*2-predicted distance probabilities in more detail (see, for example, Brown *et al.*, 2024 ▸) can flag such regions directly, thereby avoiding these rare mispredictions.

In summary, our method, which is map-independent and orthogonal to the prevalent validation software, effectively pinpoints register errors in large numbers of mid-resolution PDB entries, illustrating the challenges facing even diligent and expert structural biologists when working on this kind of target. Notably, and unlike other validation methods, our method provides a suggested correction to the register for putative errors. Finally, with a simple adaptation to use C^α^ rather than C^β^ atoms in the definition of contacts, we expect that our method could answer the recent call from the PDB cryo-EM Data Archiving and Validation Group (Kleywegt *et al.*, 2024 ▸) for methods to validate C^α^-only structures. We hope that the availability of our method through *CCP*4 (Agirre *et al.*, 2023 ▸) will contribute to detecting errors before deposition in the PDB.

## Supplementary Material

Supplementary figures and key to Supplementary Table S1. DOI: 10.1107/S2052252524009114/lz5074sup1.pdf

Supplementary Table S1: a list of putative register errors. DOI: 10.1107/S2052252524009114/lz5074sup2.xlsx

## Data Availability

The set of predicted register errors is available in Supplementary Table S1. *conkit-validate* is distributed with the *CCP*4 software suite (Agirre *et al.*, 2023 ▸).
